# Numerical Simulation of Friction Stir Welding of Dissimilar Al/Mg Alloys Using Coupled Level Set and Volume of Fluid Method

**DOI:** 10.3390/ma17123014

**Published:** 2024-06-19

**Authors:** Guanlan Zhang, Jinqiang Gao, Chuansong Wu

**Affiliations:** MOE Key Laboratory for Liquid-Solid Structure Evolution and Materials Processing, Institute of Materials Joining, Shandong University, Jinan 250061, China; 202014165@mail.sdu.edu.cn (G.Z.); jqg@sdu.edu.cn (J.G.)

**Keywords:** friction stir welding, dissimilar alloys, material mixing, level set, volume of fluid, numerical simulation

## Abstract

The coupled level set and volume of fluid (CLSVOF) method is proposed to simulate the material distribution and physical properties during dissimilar aluminum/magnesium friction stir welding (FSW) process more accurately. Combined with a computational fluid dynamics model, the FSW process is numerically simulated and the heat transfer and material flow are analyzed. The results show that heat transfer and material flow have great influence on the Al/Mg bonding. In order to verify the accuracy of the model, the calculated results based on different methods are compared with the experimental results, and the Al/Mg interface simulated by the CLSVOF method is in better agreement with the experimental results. Finally, the material distribution and interface evolution near the tool at different times were studied based on the CLSVOF method.

## 1. Introduction

The complex structure composed of different lightweight materials (aluminum alloy and magnesium alloy) has attracted wide attention in the aerospace, automobile, and other sectors [1,2]. However, the large differences in the crystal structure and physical properties between the two metals make it difficult to join them together by using fusion welding processes. Friction stir welding (FSW), as a solid-state process, is suitable for joining dissimilar materials, and plentiful experiments have been carried out [3,4,5]. To understand the complex underlying physics in FSW process of dissimilar Al/Mg alloys, the combination of numerical simulation and experimental verification is the most economical and effective method.

In previous studies, computational fluid dynamics (CFD) was a common means to simulate the heat transfer and material flow in the dissimilar FSW process, where multiphase flow theory and the volume of fluid (VOF) method were used to describe the mixing and distribution of dissimilar materials [6,7]. VOF is a single-fluid algorithm derived from continuous equations with the property of mass conservation [8]. However, the discontinuity of the function defined by the VOF method makes it difficult to obtain the exact interface curvature and the physical properties of materials near the interface, which reduces the stability of the calculation process and leads to poor accuracy of interface reproduction.

The level set method proposed by Osher et al. [9] is another way of solving two-phase fluid flow. The method can track the interface changes by calculating the zero-level set at the new time step. Compared with the VOF method, it has higher accuracy in the interface calculation due to its smooth continuity. Tomashchuk et al. [10] took the electron beam welding of copper/steel as an example and used the level set method to solve the motion equation of the interface between two metals. The position of the interface on a two-dimensional plane can be easily obtained and can approximately reproduce the weld morphology of two metals. Kesharwani et al. [11] numerically analyzed the FSW process of Al 6061-T6 with Al_2_O_3_ particles and predicted the mixing of the two fluids using the level set method. Sadeghian et al. [12] used the level set method to simulate weld morphology and predicted dissimilar material flow behaviors in the aluminum/steel FSW process. The results proved that the level set method can accurately predict material flow behaviors. However, in the process of solving the level set equation, there is a small amount of mass loss/increase at each time step due to the dissipation effect in the numerical method, and the error accumulation over time leads to the gradual distortion and even ambiguity of the solved moving interface [13]. Combining the advantages of mass conservation of the VOF method and the smooth continuity of the level set method, Sussman and Puckett [14] proposed a coupled level set and volume of fluid (CLSVOF) method to calculate two-phase flow. However, the CLSVOF method is mostly used in gas dynamics [15,16] and is rarely used in welding process, especially in the FSW process.

In this work, the CLSVOF method, combined with a three-dimensional multiphase CFD model, is employed to study the dissimilar Al/Mg FSW process. The material distribution on different horizontal planes is predicted by the level set function and the smooth material physical properties are obtained near the Al/Mg interface. The heat transfer and material flow behavior of the FSW process simulated based on the VOF and CLSVOF methods are compared and analyzed. The experimental results are used to verify the accuracy of the model. Finally, the material distribution and interface evolution near the tool at different times are studied based on the CLSVOF method.

## 2. Model Formulation

### 2.1. Governing Equations

A CFD model is established to predict the heat transfer and material flow behavior of the dissimilar Al/Mg FSW process. The Al and Mg alloys experiencing severe plastic deformation around the FSW tool are treated as an incompressible non-Newtonian visco-plastic flow, where the Al alloy is the primary phase and the Mg alloy is the secondary phase. The heat transfer and fluid flow are governed by the following equations:

(1) $$\frac{\partial \rho_{m}}{\partial t}+\rho_{m}\nabla \cdot \vec{V}=0$$

(2) $$\rho_{m}\left[\frac{\partial \vec{V}}{\partial t}+\left(\vec{V}\cdot \nabla \right)\vec{V}\right]=-\nabla P+\mu_{s,m}\nabla^{2}\vec{V}+{\vec{F}}_{\sigma }$$

(3) $$\rho_{m}\;c_{p,m}\left(\frac{\partial T}{\partial t}+\vec{V}\cdot \nabla T\right)=\nabla \cdot (\lambda_{m}\nabla T)+S_{v,m}$$

where $\rho_{m}$ is the fluid density, $\vec{V}$ is the flow velocity vector, t is the time, P is the static pressure, ${\vec{F}}_{\sigma }$ is the interacting force between two phases and is zero except for the cell at the interface, $c_{p,m}$ is the heat capacity, $\lambda_{m}$ is the thermal conductivity, $S_{v,m}$ is the viscous dissipation heat generated by the plastic deformation material around the tool, which is related to the material viscosity and strain rate. The viscosity, $\mu_{s,m}$, can be obtained as follows:

(4) $$\mu_{s,m}=\frac{\sigma_{s,m}}{3\dot{\varepsilon }}$$

where $\dot{\varepsilon }$ is the strain rate, and $\sigma_{s,m}$ is the flow stress. Other physical properties of base materials, constitutive equation parameters related to flow stress, and boundary condition settings have been comprehensively described in a previous study [17].

### 2.2. VOF Method

The VOF method is commonly used to model the dissimilar FSW process, where the interaction between different fluids is expressed by the volume fraction of different phases in a cell. The sum of the volume fractions of each phase in a cell is equal to 1 [18]:

(5) $$\alpha_{\mathrm{Al}}+\alpha_{\mathrm{Mg}}=1$$

where α is the volume fraction, and the subscripts Al and Mg indicate Al alloy and Mg alloy, respectively. Therefore, we just need to solve for the volume fraction of one phase to obtain the two-phase distribution. The volume fraction of the secondary phase is controlled by the continuity equation [19]:

(6) $$\nabla \cdot \left(\alpha_{\mathrm{Mg}}\rho_{\mathrm{Mg}}{\vec{V}}_{\mathrm{Mg}}\right)={\dot{m}}_{\mathrm{AlMg}}-{\dot{m}}_{\mathrm{MgAl}}$$

where ${\vec{V}}_{\mathrm{Mg}}$ is the flow velocity of Mg alloy, $\rho_{\mathrm{Mg}}$ is the density of Mg alloy, and ${\dot{m}}_{\mathrm{AlMg}}$ and ${\dot{m}}_{\mathrm{MgAl}}$ are the mass transfer from Al to Mg and from Mg to Al in unit volume and in unit time, respectively.

The physical properties of each cell $M_{m}$, such as density and viscosity, can be calculated by the volume fraction of the primary phase:

(7) $$M_{m}=M_{\mathrm{Al}}\alpha_{\mathrm{Al}}+M_{\mathrm{Mg}}\left(1-\alpha_{\mathrm{Al}}\right)$$

The interacting force between two phases is described by the continuous surface tension (CSF) model [20]:

(8) $${\vec{F}}_{\sigma }=\sigma_{\mathrm{Al}\text{-}\mathrm{Mg}}\kappa \cdot \vec{n}\delta_{\Gamma }$$

where $\sigma_{\mathrm{Al}-\mathrm{Mg}}$ is the surface tension coefficient, and the normal vector $\vec{n}$ and interface curvature κ are calculated from the volume fraction ($\vec{n}=\nabla \alpha /\left|\nabla \alpha \right|$ and $\kappa =-\nabla \cdot \vec{n}$). The Dirac function $\delta_{\Gamma }$ is approximately |∇α| in the sense of distribution.

### 2.3. CLSVOF Method

Since the volume fraction is a step function, it is difficult to obtain the exact physical properties near the interface, so the level set method is introduced to trace the two-phase moving interface. The level set function ϕ(x,y,z,t) is always the symbolic distance function from the point (x,y,z) to the interface, and the interface is the zero-level set [21]:

(9) $$\phi \left(x,y,z,t\right)=\{\begin{array}{ccc} >0 & & Fluid\begin{array}{c} 1 \end{array}\\ 0 & & Interface\\ <0 & & Fluid\begin{array}{c} 2 \end{array} \end{array}$$

At any time t, the point on the interface satisfies:

(10) $$\frac{\partial \phi }{\partial t}+\vec{V}\cdot \nabla \phi =0$$

By solving the above equation to determine the zero-level set, the interface tracking is realized. Equation (10) is the fundamental equation of the level set method.

However, the level set function no longer remains the defined symbolic distance function after several solving time steps, which will lose the meaning that the zero-level set is the moving interface. Consequently, the level set function needs to be reinitialized at each time step. The reinitialization equation can be expressed as [22,23]:

(11) $$\{\begin{array}{c} \frac{\partial \phi }{\partial \tau }=S\left(\phi_{0}\right)\left(1-\left|\nabla \phi \right|\right)\\ \phi \left(x,y,z,0\right)=\phi_{0}\left(x,y,z\right) \end{array}$$

where τ is the virtual iteration time, which is not equal to the actual time t; $S\left(\phi_{0}\right)$ is a symbolic function; and $\phi_{0}$ is the initial value of the level set function.

During the process of reinitialization, the initial value is obtained from the volume fraction [20,24]:

(12) $$\phi_{0}=\left(2\alpha_{\mathrm{Al}}-1\right)\cdot m\Delta h$$

where Δh is the mesh size, and m is taken to correct the initial ϕ so that the value is close to the correct distance to the interface.

In order to avoid the instability of the calculation process, the symbolic function can be smooth [24,25]:

(13) $$S\left(\phi_{0}\right)=\frac{\phi_{0}}{\sqrt{{\phi_{0}}^{2}+{\left(\Delta h\right)}^{2}}}$$

A smooth function H(ϕ) related to level set function is defined to represent the material distribution; the value ranges from 0 (Mg alloy) to 1 (Al alloy), which can be expressed as [26]:

(14) $$H\left(\phi \right)=\{\begin{array}{cc} \begin{array}{ccc} 0 & & if\phi <-\gamma \\ \frac{1}{2}\left[1+\frac{\phi }{\gamma }+\frac{1}{\pi }\sin\left(\frac{\pi \phi }{\gamma }\right)\right] & & if\left|\phi \right|\leq \gamma \\ 1 & & if\phi >\gamma \end{array} & \end{array}$$

where γ is an adjustable parameter used to indicate the thickness of the Al/Mg interface.

Therefore, in the CLSVOF method, physical property parameters can be expressed as:

(15) $$M_{m}=M_{\mathrm{Al}}H\left(\phi \right)+M_{\mathrm{Mg}}\left(1-H\left(\phi \right)\right)$$

In addition, the normal vector in Equation (8) is recalculated by the level set function $\vec{n}=\nabla \phi /\left|\nabla \phi \right|$, which provides a more accurate interface curvature $\kappa =-\nabla \cdot \vec{n}$ than the VOF-based value. $\delta_{\Gamma }$ is redefined as in [27]:

(16) $$\delta_{\Gamma }=\{\begin{array}{ccc} 0 & & \left|\phi \right|>\gamma \\ \frac{1}{2\gamma }\left[1+\cos\left(\frac{\pi \phi }{\gamma }\right)\right] & & \left|\phi \right|<\gamma \end{array}$$

### 2.4. Implementation of CLSVOF

ANSYS FLUENT 16.0 software has a perfect and powerful calculation function for flow field and can add some conditions with user defined functions so that it has great flexibility. Therefore, the VOF model provided by the software is selected in this study, and the model is modified on this basis to realize the application of the CLSVOF method in the numerical simulation of the Al/Mg dissimilar FSW so as to improve the reliability and accuracy of the calculation.

Based on the two-phase incompressibility assumption, the non-conserved level set equation can be conserved:

(17) $$\frac{\partial \phi }{\partial t}+\nabla \cdot \left(\phi \vec{V}\right)=0$$

Set the diffusion coefficient and source term of the standard user defined scalar (UDS) equation in FLUENT to zero, and the UDS equation can be expressed as:

(18) $$\frac{\partial \left(\rho \phi \right)}{\partial t}+\nabla \cdot \left(\rho \phi \vec{V}\right)=0$$

As can be seen from the above, by returning the unsteady term and convection term without density by the Fluent user-defined function, Equation (18) can be converted to Equation (17) so that the solution of the UDS equation is the value of the level set function.

In the reinitialization equation, Equation (11), the steady-state solution of the equation, converges to |∇ϕ|=1. The Godunov–Hamiltonian function is adopted to represent the gradient in the equation [28]:

(19) $$G=\{\begin{array}{cc} \sqrt{\max\left({\left(a^{+}\right)}^{2},{\left(b^{-}\right)}^{2}\right)+\max\left({\left(c^{+}\right)}^{2},{\left(d^{-}\right)}^{2}\right)+\max\left({\left(e^{+}\right)}^{2},{\left(f^{-}\right)}^{2}\right)} & \phi >0\\ \sqrt{\max\left({\left(a^{-}\right)}^{2},{\left(b^{+}\right)}^{2}\right)+\max\left({\left(c^{-}\right)}^{2},{\left(d^{+}\right)}^{2}\right)+\max\left({\left(e^{-}\right)}^{2},{\left(f^{+}\right)}^{2}\right)} & \phi <0\\ 1 & \phi =0 \end{array}$$

where $a^{+}=\max\left(\phi_{x}^{-},0\right)$, $a^{-}=\min\left(\phi_{x}^{-},0\right)$, $b^{+}=\max\left(\phi_{x}^{+},0\right)$ and $b^{-}=\min\left(\phi_{x}^{+},0\right)$, $\phi_{x}^{-}$, and $\phi_{x}^{+}$ are the left and right derivatives in the X direction, respectively. The same method can be used to obtain $c^{+}-f^{-}$ in the Y and Z directions.

**Figure 1:**
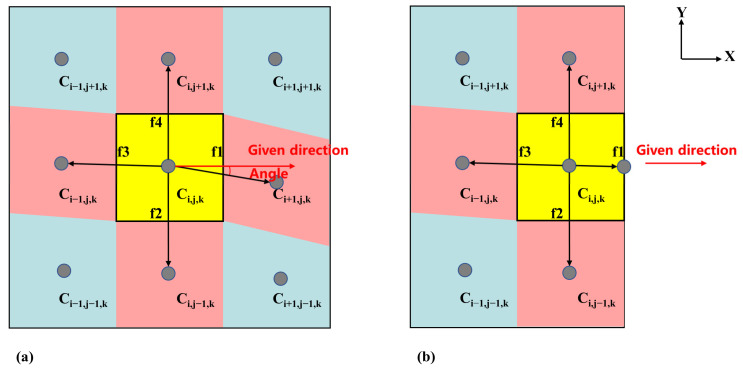
Diagram of the current cell and its adjacent cell, where the yellow cell represents the current cell, the red cell represents its adjacent cell and the blue cell represents the other cell. (**a**) Internal cell and (**b**) boundary cell.

At present, the fifth-order weighted essentially non-oscillatory (WENO) scheme is the most efficient and accurate computing scheme for solving spatial derivatives [29]. The main idea of the fifth-order WENO scheme is that for a cell i, to calculate $\phi_{x}^{-}$, six left-sided nodes in the X direction $\left\{\phi_{i-3},\phi_{i-2},\phi_{i-1},\phi_{i},\phi_{i+1},\phi_{i+2}\right\}$ are selected to build the interpolation. Similarly, six right-sided nodes $\left\{\phi_{i-2},\phi_{i-1},\phi_{i},\phi_{i+1},\phi_{i+2},\phi_{i+3}\right\}$ are selected to calculate $\phi_{x}^{-}$. To find a neighbor cell in a given direction of a cell in FLUENT, we need to write a function to perform this manually. As shown in Figure 1, if we want to obtain the adjacent cell $C_{i+1,j,k}$ in the positive X direction of the cell $C_{i,j,k}$, we need to connect the current cell center to all neighboring cell centers as vectors and then find the smallest angle between these vectors and the direction (1,0,0). The smallest angle-corresponding cell is the adjacent cell we need (Figure 1a). If the angle corresponds to the boundary surface, there is no adjacent cell (Figure 1b). By specifying different directions, adjacent cells in different directions can be obtained.

When calculating $\phi_{x}^{-}$, we set:

(20) $$\begin{array}{l} e_{1}=\frac{\phi_{i-2,j,k}-\phi_{i-3,j,k}}{x_{i-2}-x_{i-3}}\\ e_{2}=\frac{\phi_{i-1,j,k}-\phi_{i-2,j,k}}{x_{i-1}-x_{i-2}}\\ e_{3}=\frac{\phi_{i,j,k}-\phi_{i-1,j,k}}{x_{i}-x_{i-1}}\\ e_{4}=\frac{\phi_{i+1,j,k}-\phi_{i,j,k}}{x_{i+1}-x_{i}}\\ e_{5}=\frac{\phi_{i+2,j,k}-\phi_{i+1,j,k}}{x_{i+2}-x_{i+1}} \end{array}$$

When calculating $\phi_{x}^{+}$, we set:

(21) $$\begin{array}{l} e_{1}=\frac{\phi_{i+3,j,k}-\phi_{i+2,j,k}}{x_{i+3}-x_{i+2}}\\ e_{2}=\frac{\phi_{i+2,j,k}-\phi_{i+1,j,k}}{x_{i+2}-x_{i+1}}\\ e_{3}=\frac{\phi_{i+1,j,k}-\phi_{i,j,k}}{x_{i+1}-x_{i}}\\ e_{4}=\frac{\phi_{i,j,k}-\phi_{i-1,j,k}}{x_{i}-x_{i-1}}\\ e_{5}=\frac{\phi_{i-2,j,k}-\phi_{i-1,j,k}}{x_{i-2}-x_{i-1}} \end{array}$$

The left and right spatial derivatives can be calculated from the following general formula:

(22) $${\left(\phi_{x}^{\pm }\right)}_{i,j,k}=w_{1}\left(\frac{e_{1}}{3}-\frac{7e_{2}}{6}+\frac{11e_{3}}{6}\right)+w_{2}\left(-\frac{e_{2}}{6}-\frac{5e_{3}}{6}+\frac{e_{4}}{3}\right)+w_{3}\left(-\frac{e_{3}}{3}-\frac{5e_{4}}{6}+\frac{e_{5}}{6}\right)$$

The weighted values $w_{1}$, $w_{2}$, and $w_{3}$ can be expressed as:

(23) $$\begin{array}{l} w_{k}=\frac{\beta_{k}}{\beta_{1}+\beta_{2}+\beta_{3}}\begin{array}{cc} & \left(k=1,2,3\right) \end{array}\\ \beta_{1}=\frac{1}{10}\frac{1}{{\left(\varpi +S_{1}\right)}^{2}}\begin{array}{cc} & \beta_{2}=\frac{6}{10}\frac{1}{{\left(\varpi +S_{2}\right)}^{2}} \end{array}\begin{array}{cc} & \beta_{3}=\frac{3}{10}\frac{1}{{\left(\varpi +S_{3}\right)}^{2}} \end{array} \end{array}$$

where ϖ is a minimal empirical constant ($\varpi =10^{-6}$), and $S_{1}$, $S_{2}$ and $S_{3}$ are defined as:

(24) $$\begin{array}{l} S_{1}=\frac{13}{12}{\left(e_{1}-2e_{2}+e_{3}\right)}^{2}+\frac{1}{4}{\left(e_{1}-4e_{2}+3e_{3}\right)}^{2}\\ S_{2}=\frac{13}{12}{\left(e_{2}-2e_{3}+e_{4}\right)}^{2}+\frac{1}{4}{\left(e_{2}-e_{4}\right)}^{2}\\ S_{3}=\frac{13}{12}{\left(e_{3}-2e_{4}+e_{5}\right)}^{2}+\frac{1}{4}{\left(3e_{3}-4e_{4}+e_{5}\right)}^{2} \end{array}$$

In a similar way, $\phi_{y}^{-}$, $\phi_{y}^{+}$, $\phi_{z}^{-}$, and $\phi_{z}^{+}$ can be calculated.

The first-order Euler method or the third-order Runge–Kutta method are usually used to solve the time derivative. Some researchers have shown that the results obtained by the first-order method are basically consistent with those obtained by the third-order method [20]. Therefore, in order to save calculation time, the time term is calculated by the first-order Euler form:

(25) $$\phi^{n}=\phi^{n-1}+\Delta \tau S\left(\phi_{0}\right)\left[1-\left|\nabla \phi^{n-1}\right|\right]$$

where n is the maximum iterations. The stable solution $\phi \left(x,y,z,\tau_{\mathrm{steady}}\right)$ of the reinitialization equation will be used for the calculation at the next time.

### 2.5. Geometric Model

**Figure 2:**
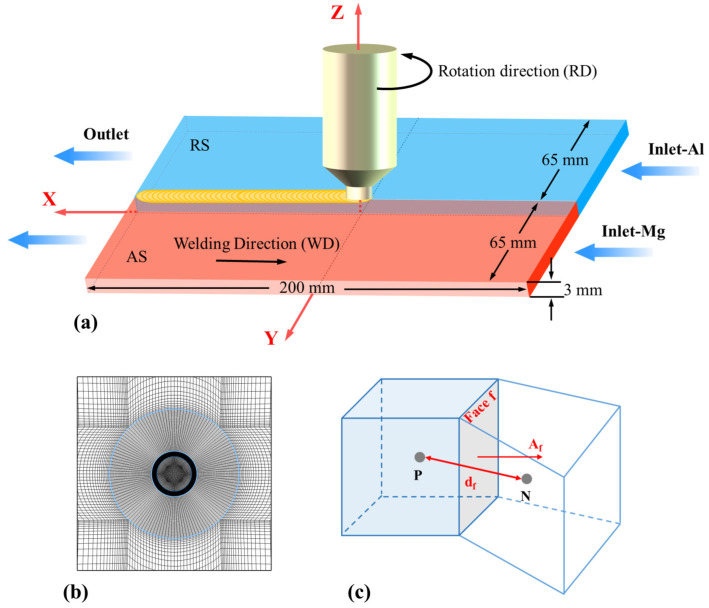
Geometric model and mesh generation. (**a**) Geometric model, (**b**) mesh near the tool, and (**c**) parameters related to mesh size.

The base metals are 6061 Al alloy and AZ31B Mg alloy sheets, with sizes of 200 mm (length) × 65 mm (width) × 3 mm (height). The chemical composition of the base materials is listed in Table 1. The FSW tool is made of H13 steel, and the diameters of the shoulder, the pin root, and pin tip are 12 mm, 4.2 mm, and 3.2 mm, respectively. The Mg alloy is placed on the advancing side (AS) and the Al alloy on the retreating side (RS), and the welding process parameters are set as the rotating speed of 800 rpm and the welding speed of 50 mm/min. The geometric features of the tool, such as pin thread, tilt angle, and shoulder plunge depth, are ignored and the CFD model of the Al/Mg dissimilar alloy FSW process is established, as shown in Figure 2a. The ICEM CFD 16.0 software is used to generate grids on the workpiece, regardless of the tool that is considered to be a rigid body. Considering the complex interaction between the tool and the workpiece, a large mesh refinement is performed near the tool and the original Al/Mg interface (Figure 2b). The mesh size is defined as the average distance between the current cell center and all adjacent cell centers:

(26) $$\Delta h=\frac{\sum_{f}^{N_{f}}\left|{\vec{n}}_{P}\cdot {\vec{A}}_{f}\right|d_{f}}{\sum_{f}^{N_{f}}\left|{\vec{n}}_{P}\cdot {\vec{A}}_{f}\right|}\begin{array}{cc} & if\left|{\vec{n}}_{P}\right| \end{array}\neq 0$$

where $N_{f}$ is the number of faces of the cell *P*, ${\vec{A}}_{f}$ is the area vector of the face *f*, $d_{f}$ is the distance between the cell center and its adjacent cell center, and ${\vec{n}}_{p}$ is the normal vector at *P*, as shown in Figure 2c.

### 2.6. Solution Algorithm

**Figure 3:**
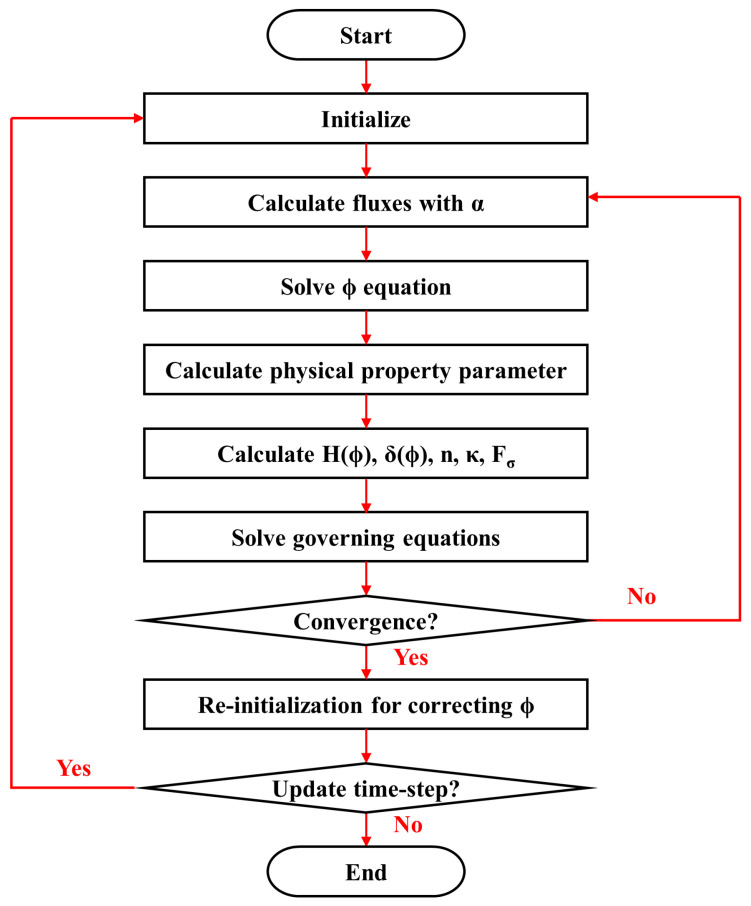
The calculation flow of CFD model based on CLSVOF method.

Figure 3 shows the calculation flow chart of the CFD model based on the CLSVOF method, and the detailed instructions are as follows:

1. Open and initialize the UDS equation to solve the level set function;
2. On the basis of the initial conditions or the value of the previous time step, initialize the physical quantities required to be solved, such as velocity, density, and level set function;
3. Solve the VOF equation, Equation (6);
4. Solve the level set equation, Equation (10);
5. Calculate relevant physical properties and store normal vector components, interface curvature, and other intermediate values;
6. Add the interacting force between two phases in the form of the source term to the momentum equation, solve the governing equations, Equations (14)–(16), and obtain an updated velocity field;
7. Assign the initial value to the level set function according to the VOF function, and use Equation (11) to reinitialize the level set function;
8. Go to the next time and repeat step 2–7.

## 3. Results and Discussion

Based on the CLSVOF method, the Al/Mg dissimilar alloy FSW process is numerically simulated, and the level set equation is solved by the transient model. Since the transient calculation takes a long time, a method combining steady state and transient state is proposed to save computing resources. Firstly, the multiphase steady-state laminar flow simulation based on the VOF method (VOF model) is carried out, and then the steady-state calculation results (velocity field, etc.) are used as the initial solution condition of level set equation to calculate the transient multiphysics field (CLSVOF model) during the dissimilar welding. When the calculation procedure is converged, the material flow and heat generation in the FSW process are extracted and analyzed. In the calculation, it is found that the level set function can reach the stable state quickly, and the transient simulation time is set to 8 s.

**Figure 4:**
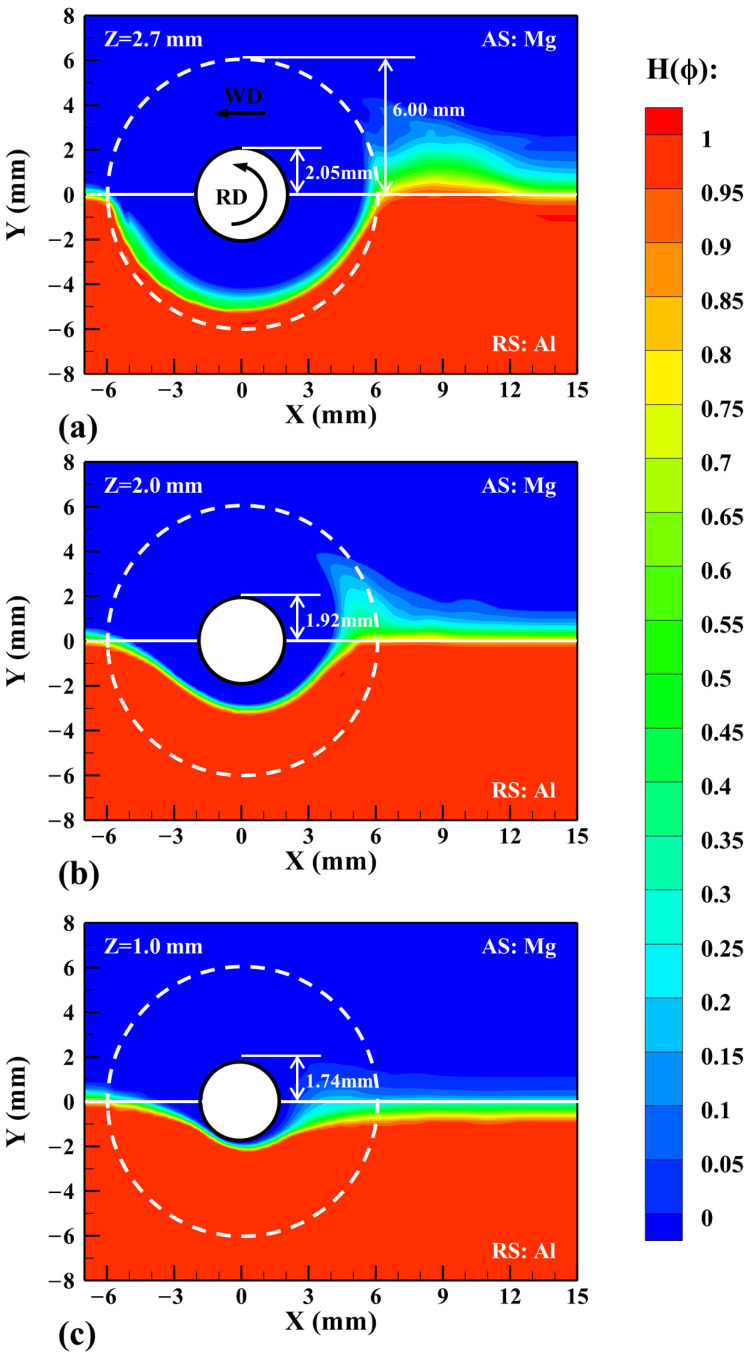
The material distribution on different horizontal planes. (**a**) Z = 2.7 mm, (**b**) Z = 2.0 mm, and (**c**) Z = 1.0 mm.

In the CLSVOF model, H(ϕ) in Equation (14) is used to represent the distribution of two materials. Figure 4 shows the material distribution calculated on different horizontal planes, where the dashed white line represents the shoulder edge and the solid white line represents the original Al/Mg abutting interface. It can be clearly seen that the Mg alloy at the AS flows to the Al at the RS and deposits at the trailing side (TS) of the tool. The maximum flow area is located near the shoulder and the flow area gradually decreases with the z-coordinate decreases. On the upper horizontal plane of the workpiece in Figure 4a, the Al alloy flows across the original abutting interface to the AS under the combined action of the shoulder and the pin, and the degree of Al/Mg bonding is better. On the lower horizontal planes of the workpiece in Figure 4b,c, the shoulder action is weakened and the pin’s action is not enough to allow more Mg alloy to flow to the RS, so the flow range is narrowed. At the same time, the distance of Al alloy moving across the original abutting interface is also reduced, and it is difficult to form a good combination between the Al and Mg alloys, which is consistent with the fact that the defects often observed in the pin’s affected zone.

**Figure 5:**
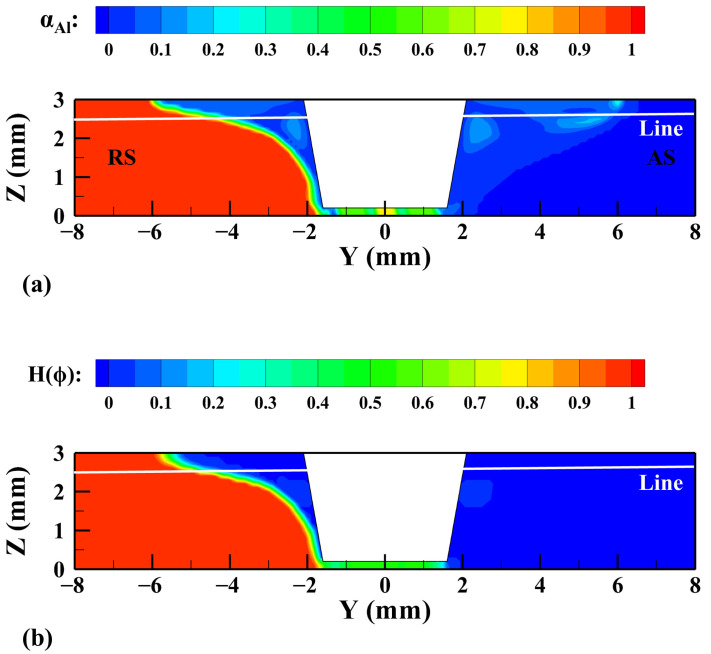
The material distribution calculated by different models on the cross-section of workpiece. X = 0 mm. (**a**) VOF and (**b**) CLSVOF.

Then, the heat transfer and material flow in the welding process are simulated based on the CLSVOF model, and the results are compared with those based on the VOF model to explore the advantages of the CLSVOF model. The first thing to understand is that the two models use the volume fraction $\alpha_{\mathrm{Al}}$ and smoothing function H(ϕ) to distinguish the Al alloy, Mg alloy, and Al/Mg mixing zone, respectively. Figure 5 shows the material distribution calculated by the two models on the cross-section of the workpiece X = 0 mm. It can be seen that compared with the calculation results of the VOF model, the two-phase interface calculated by the CLSVOF model is clearer, and a part of the Al alloy replaces the Mg alloy on the RS, so the Al/Mg interface shows a trend of concentration towards the middle position.

**Figure 6:**
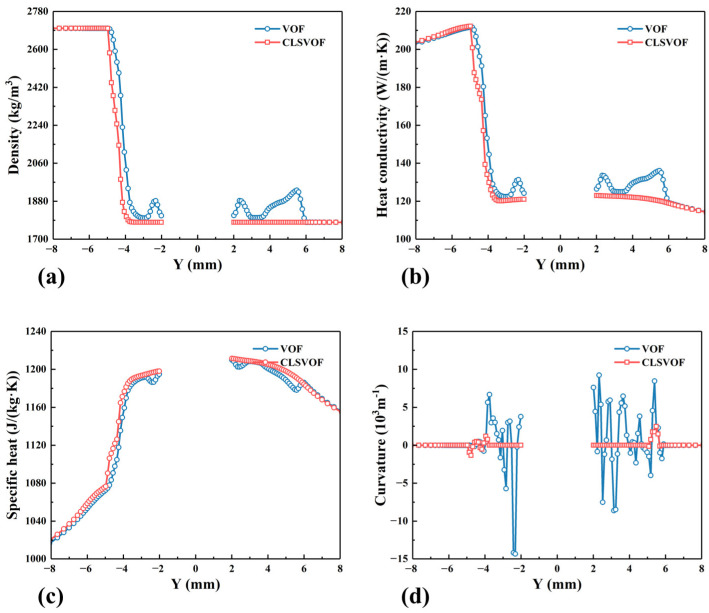
Physical property curves near the interface calculated by different models. (**a**) Density, (**b**) thermal conductivity, (**c**) heat capacity, and (**d**) interface curvature.

According to Equations (7) and (15), the distribution of materials will affect the physical properties of materials and then affect the heat generation and material flow in the welding process. The line Z = 2.5 mm is selected on the cross section in Figure 5, and the physical properties calculated by different models on the path are extracted, as shown in Figure 6. As can be seen from the figure, the calculated results of the VOF model vary greatly in the whole extraction region and the oscillations of the interface curvature are also obvious, which may adversely affect the stability of the calculation process, while the physical property curves calculated by the CLSVOF model are relatively smooth and vary greatly only near the interface.

**Figure 7:**
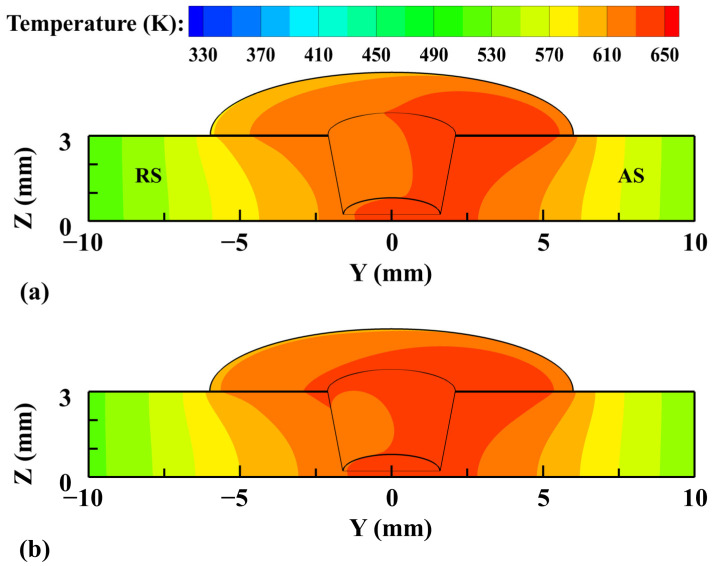
The calculated temperature field at the cross-section X = 0 mm and tool/workpiece interface for different models. (**a**) VOF and (**b**) CLSVOF.

Figure 7 shows the temperature field on the workpiece predicted by different models. The results show that the temperature distribution is obviously asymmetrical due to the different materials on both sides of the tool. In general, the distribution of the two models is basically the same. The temperature at the AS is higher than that at the RS. Taking the tool axis as the center line, the temperature calculated by the CLSVOF model is slightly higher than that in the VOF model at the same radial position, especially at the RS.

**Figure 8:**
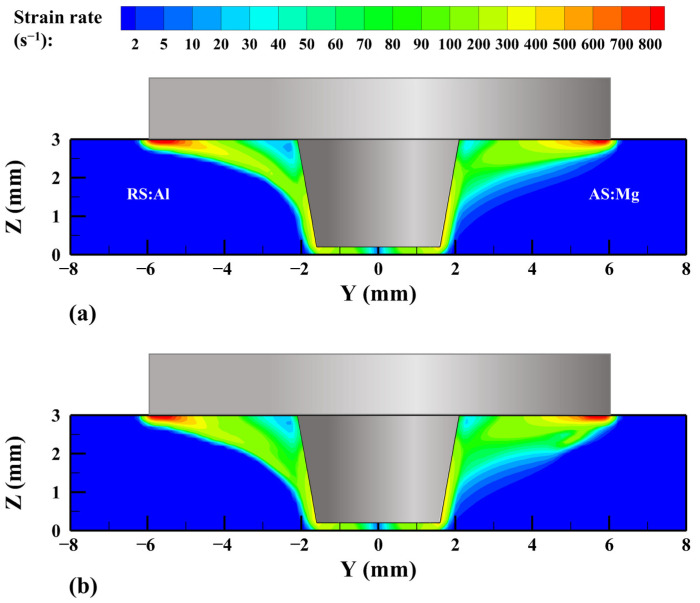
The material strain rate at cross-section of workpiece X = 0 mm. (**a**) VOF and (**b**) CLSVOF.

The variation in temperature has a significant effect on the material strain rate near the tool. Figure 8 shows the strain rate on the cross-section X = 0 mm. The closer to the pin root, the lower the strain rate of the materials. The strain rate increases gradually from the pin root to the pin bottom and the shoulder edge, reaching the maximum at the edge of the shoulder. This is because in the upper part of the workpiece, the material is subject to the combined action of the shoulder and the pin, and the material flow is more intense than that in the middle and lower part of the workpiece. At the same time, the higher temperature promoting the material softening results in a smaller strain rate at the pin root, and the lower temperature and the larger material plastic deformation make the strain rate larger near the edge of the shoulder. With the decrease in the z-coordinate, the action of the shoulder is weakened, and the temperature and plastic deformation of the material are reduced, which affects the strain rate in the thickness direction.

**Figure 9:**
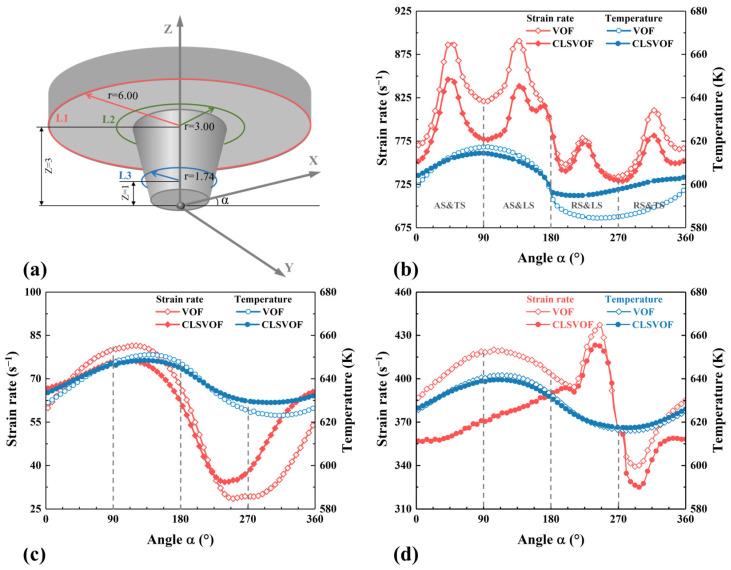
The strain rate and temperature calculated by different models. (**a**) Extraction path diagram, (**b**) L1, (**c**) L2, and (**d**) L3.

In order to better describe the relationship between temperature and strain rate, three paths, L1, L2, and L3, are selected at the shoulder bottom and the pin side, where α is the included angle between the point of the tool axis and X positive direction, as shown in Figure 9a. And the temperature and strain rate on these three paths were extracted. In the shoulder affected zone in Figure 9b,c, the higher temperature near the pin causes the material to be sufficiently softened so that the strain rate of the material there is lower. With the distance away from the tool axis increasing, the temperature gradually decreases, while the strain rate changes in the opposite trend, and the material temperature and strain rate at the AS are greater than those at the RS. The thermal conductivity of the Mg alloy is lower than that of the Al alloy, and the material temperature flowing from the lead side (LS) of the tool to the RS is lower, so the material temperature at the AS is higher. The material is stretched from the AS to the RS by the shoulder action, and the material flow is stronger at the AS, so that the strain rate is also greater. Compared with the VOF model, the Al/Mg interface in the CLSVOF model is more concentrated, resulting in more Al alloy at the RS and more Mg alloy at the AS. Thus, the material temperature is higher at the RS and lower at the AS in the CLSVOF model. In the pin’s affected zone in Figure 9d, the shear effect of the pin on the material is not as obvious as that of the shoulder, and the temperature is not as high as that of the pin root, so the strain rate on the pin side is between that on the shoulder edge and that on the pin root.

**Figure 10:**
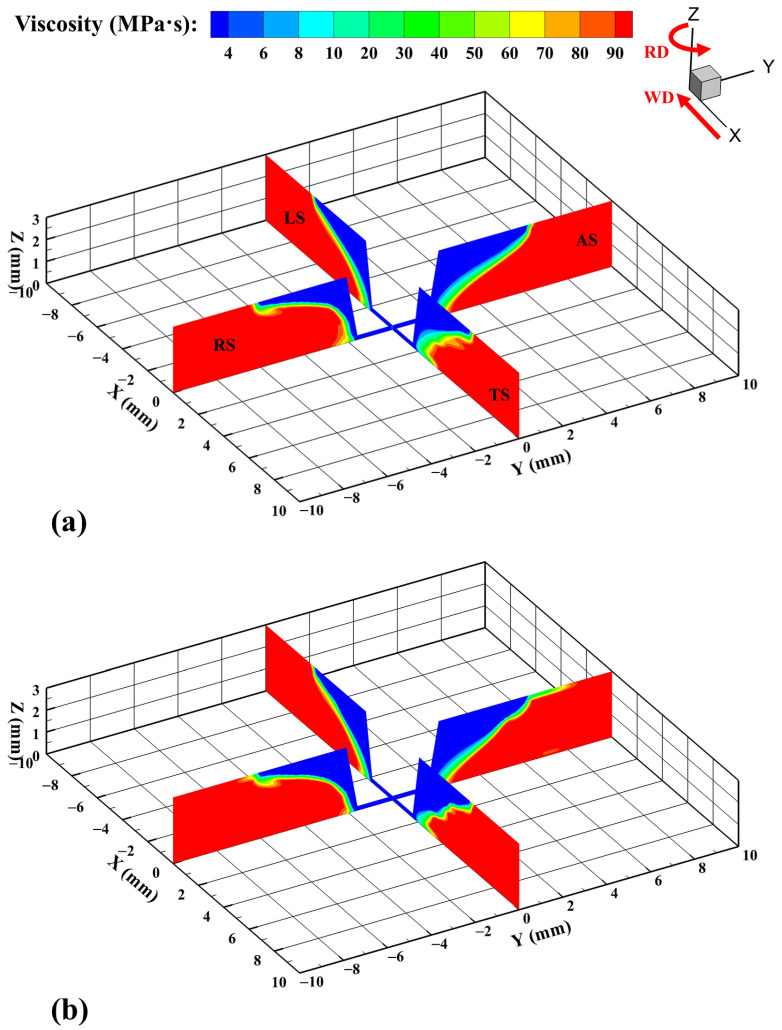
Material viscosity distribution on the transverse cross-section X = 0 mm and longitudinal cross-section Y = 0 mm. (**a**) VOF and (**b**) CLSVOF.

Material viscosity is closely related to temperature and strain rate, and temperature plays a major role. Figure 10 shows the material viscosity distribution calculated by different models. The temperature difference between the two models is mainly in the high temperature region near the tool, while the higher temperature makes the material viscosity near the tool the lowest in the whole region, so it is difficult to observe the difference in the low viscosity region. A similar result for different models is that the higher temperature and strain rate at the AS results in a larger low-viscosity region than that at the RS.

**Figure 11:**
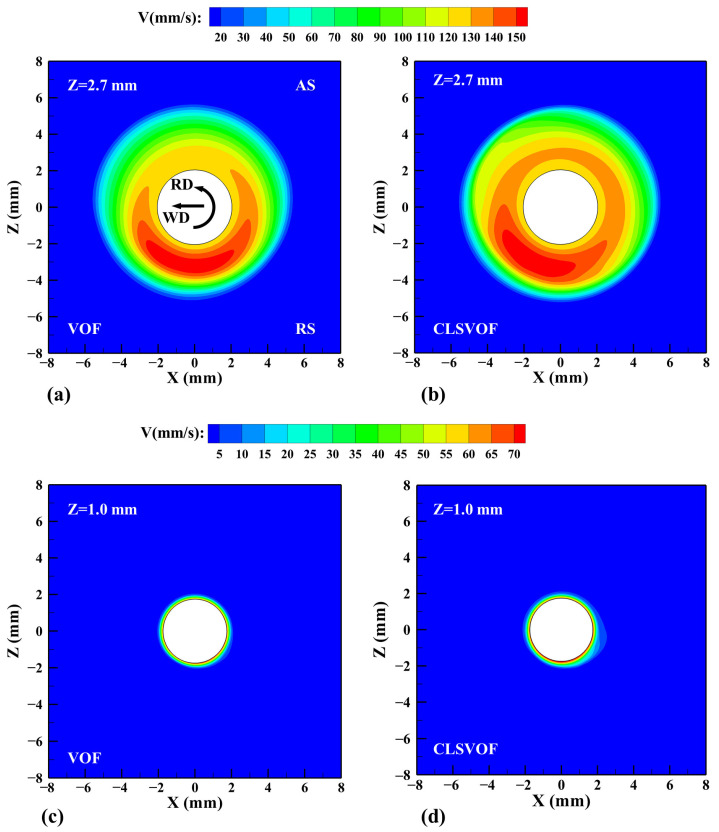
The material flow velocity on Z = 2.7 mm and Z = 1.0 mm horizontal planes. (**a**) VOF, (**b**) CLSVOF, (**c**) VOF, and (**d**) CLSVOF.

Figure 11 shows the material flow velocity on the Z = 2.7 mm and Z = 1.0 mm horizontal planes. It is obvious that the velocity at the RS is greater than that at the AS. This is because the friction direction of the tool on the material at the AS is opposite to the welding direction. When the tool moves forward, the friction impedes the material flow, making the material flow slower, and the material flow at the RS is exactly opposite to that at the AS. In the CLSVOF model, the higher temperature at the RS makes the material flow velocity larger than the result of the VOF model. On the plane near the shoulder (Z = 2.7 mm), due to the combined action of the shoulder and the pin, the flow range of the material is larger. On the plane near the pin bottom (Z = 1.0 mm), the action of the shoulder is weakened, and the flow range of the material only appears in a thin layer near the pin. The material flow velocity determines the material distribution in the welding process, which provides effective information on the formation of internal defects. The higher material velocity makes more Al alloy transfer into the Mg alloy, which contributes to the formation of an interlocking structure between Al and Mg and plays a positive role in the final weld performance. On the lower plane, the smaller material flow speed has a weak tensile/compression effect on the Al alloy, resulting in insufficient material flow, and defects are easily formed at these locations.

**Figure 12:**
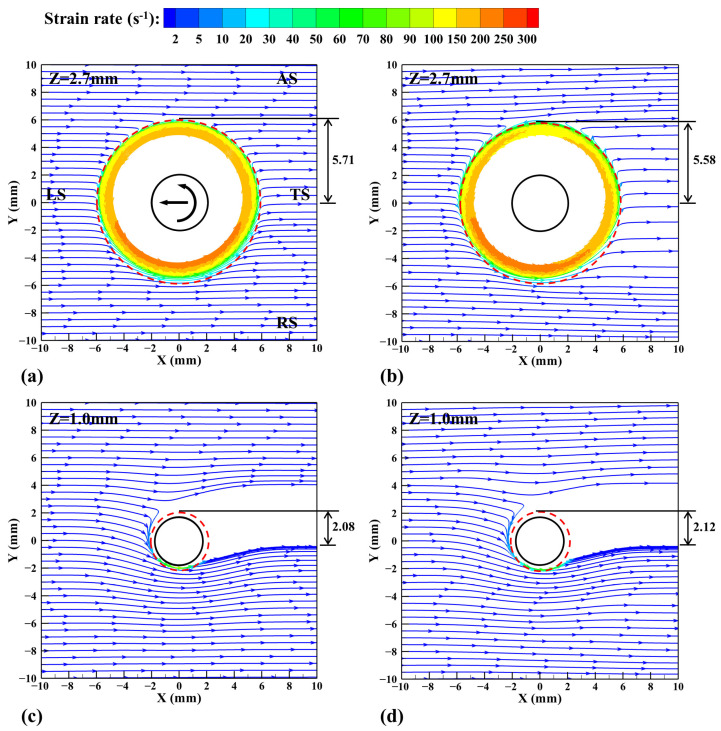
Material flow streamlines on the Z = 2.7 mm and Z = 1.0 mm horizontal planes, where blue arrows represent the material flow direction and red dashed lines represent the plastic deformation regions. (**a**) VOF, (**b**) CLSVOF, (**c**) VOF, and (**d**) CLSVOF.

Then the material flow streamlines are drawn on two horizontal planes and colored according to the strain rate, as shown in Figure 12. The plastic deformation region in the shear layer is determined by the isoline with strain rate of 2 s^−1^ and marked by the red dashed line. It can be clearly seen that the plastic deformation degree of the material in the shoulder’s affected zone is much larger than that in the pin affected zone. In the CLSVOF model, the material at the LS has a larger strain rate. Under the action of the shoulder, the material is stretched at the AS and squeezed at the RS, the flow lines deflect, and the strain rate gradually decreases. When the strain rate reduces to a certain extent, the streamlines no longer deviate, and the material flows to the TS and deposits in the same transverse position.

**Figure 13:**
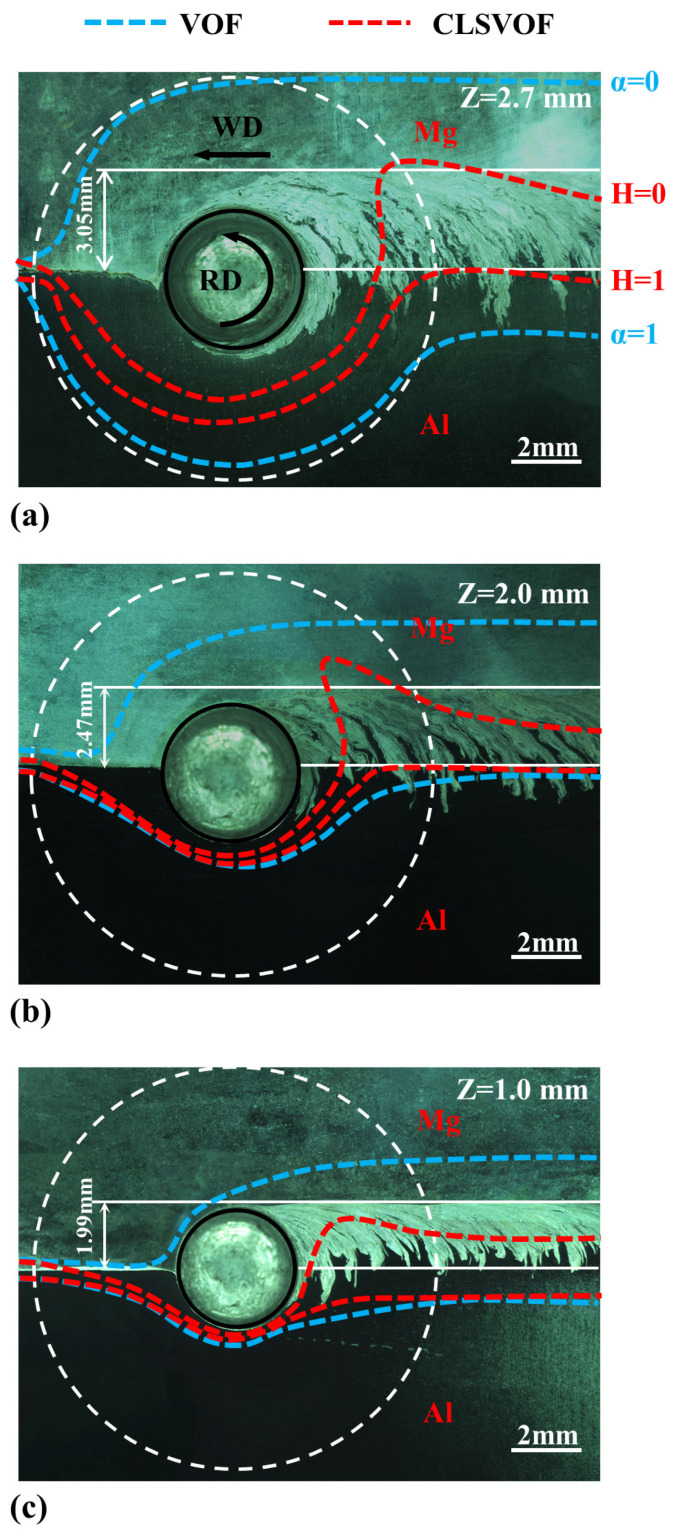
Macroscopic metallographic diagrams of weld on different horizontal planes, where the blue and red dashed lines are used to mark the Al/Mg interface calculated by the VOF and CLSVOF models, respectively. (**a**) Z = 2.7 mm, (**b**) Z = 2.0 mm, and (**c**) Z = 1.0 mm.

Figure 13 shows macrographs of welds on different horizontal planes. The area around and behind the exit hole is selected for observation. It can be seen that from the top to the bottom of the workpiece, the degree of Al/Mg bonding interface is decreasing, and the distance Al alloy penetrates into the Mg alloy across the original abutting interface is smaller. There are a large number of Al/Mg interpenetration structures near the Al/Mg bonding interface, most of which are Al alloy strips in Mg alloy, and the bonding interface is zigzag. Therefore, the region where the volume fraction $\alpha_{\mathrm{Al}}$ and smoothing function H(ϕ) vary between 0 and 1 is taken as the Al/Mg interface calculated by the VOF and CLSVOF models, respectively. They are marked by blue and red dashed lines. Previous research [30] shows that on the horizontal section around the exit hole, the material is deposited behind the pin, but as long as it is still within the contour range of the shoulder, it will still be subjected to the thermal action from the shoulder, and the material will continue to flow. Only outside the contour range of the shoulder can the material stop flowing and complete the deposition process. Therefore, the calculation results outside the shoulder profile (Al/Mg bonding interface is basically stable) are compared with the experimental results. The CLSVOF model calculates more concentrated interface zone and could better describe the Al/Mg bonding interface of the stable region. Although the interface near the exit hole is not consistent with the experimental results, with the decrease in height of the horizontal plane, the Al/Mg interface is closer and closer to the exit hole, which is consistent with the change trend of the actual interface and can qualitatively describe the interface change around the exit hole to a certain extent.

**Figure 14:**
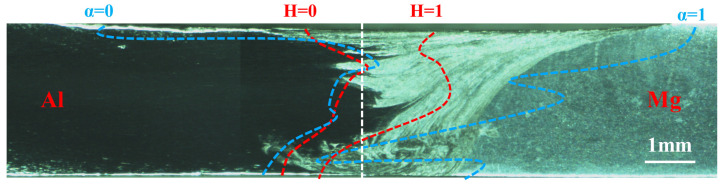
Metallographic structure of the joint in cross section (X = 30 mm).

Similarly, the calculated results of the two models are compared with the metallographic structure of the joint in cross-section, as shown in Figure 14. It can also be seen that the more concentrated interface calculated by the CLSVOF model can better describe the stable region of the Al/Mg bonding interface.

**Figure 15:**
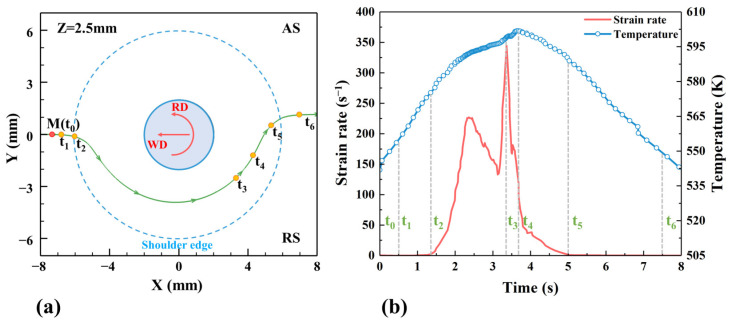
Material flow path (**a**) and its temperature and strain rate changes at point M (**b**), where the green line is the flow path and the blue dashed line is the shoulder edge.

In order to better understand the material distribution near the tool, taking the material at a point M (−7, 0, 2.5) in the LS of the shoulder as an example, the material flow path is extracted. Different positions on the path represent different moments, and the initial position represents time t_0_. Six special times, t_1_-t_6_, were also selected, as shown in Figure 15a. Figure 15b shows the curve of material strain rate and temperature with time. At time t_1_, the material does not deform, and from time t_2_, the material begins to deform plastically under the action of the shoulder ($\dot{\varepsilon }\geq 2s^{-1}$). With the increase in time, the temperature and strain rate increase, and the strain rate reaches the maximum at time t_3_. Subsequently, the strain rate began to decrease, and the temperature continued to rise and reached a peak at time t_4_. Then, the strain rate and temperature both decrease and the material plastic deformation ends at time t_5_ ($\dot{\varepsilon }<2s^{-1}$). Afterwards, the material keeps flowing under the action of the shoulder and is deposited outside the profile of the shoulder at time t_6_.

**Figure 16:**
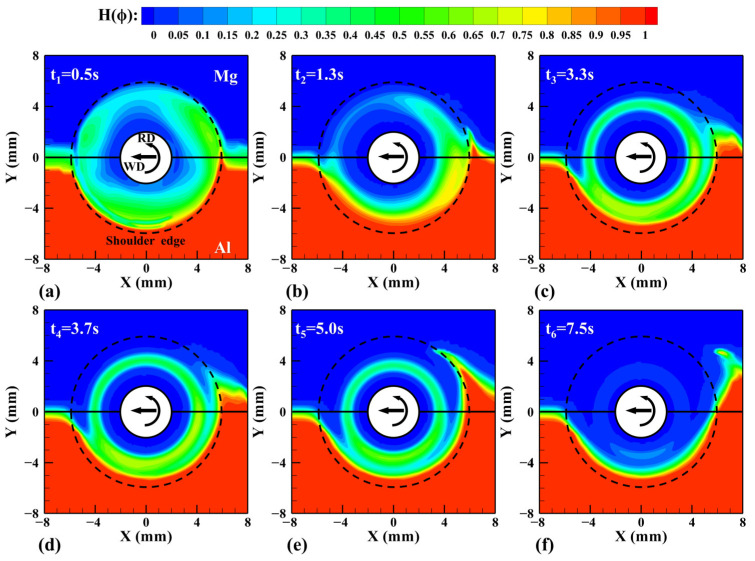
Material distribution predicted by CLSVOF method on the horizontal plane Z = 2.5 mm at different times. (**a**) t_1_, (**b**) t_2_, (**c**) t_3_, (**d**) t_4_, (**e**) t_5_, and (**f**) t_6_.

Figure 16 shows the material distribution on the horizontal plane Z = 2.5 mm at different times in Figure 15. In the initial stage of FSW, the Al alloy and Mg alloy are violently mixed under the action of high-speed rotation of the tool. And then the tool moves along the welding direction, the material at the LS begins to deform at time t_2_, and the Mg alloy at the AS flows to the material at the RS under the shearing action of the tool. Between time t_3_ and time t_4_, the higher strain rate and temperature make the plastic deformation of the material reach the maximum, and the Al alloy easily loses continuity, a part of the material is migrated to the TS of the tool, and the other part of the material is migrated near the pin and makes a downward spiral movement under the shear and extrusion of the pin. At the time t_5_, the material ends its deformation, but under the thermal action of the tool, the material will still flow to a certain extent. Some materials flow to the TS with other materials and are deposited, and some plastic materials continue to move downward until the tool bottom, gradually forming a stable and continuous two-phase interface.

## 4. Conclusions

1. The coupled level set and volume of fluid (CLSVOF) method is proposed and the method can not only maintain the conservation of mass but also accurately calculate the material physical properties near the interface. The material mixing and distribution in the Al/Mg dissimilar FSW are studied by using the CLSVOF method. In the upper part of the workpiece, the distance of the Al alloy moving across the original abutting surface is larger than that in the middle and lower part of the workpiece, and the degree of Al/Mg bonding is better.
2. A comparative analysis is conducted on the heat transfer and material flow in the Al/Mg dissimilar FSW process by using the VOF model and CLSVOF model. The heat transfer and material flow play important roles on the mechanical locking between Al and Mg alloys. At the lower part of the workpiece, low heat and poor material fluidity result in weak bonding, making it easy for defects to occur there.
3. In order to verify the accuracy of the model, the predicted weld morphologies are compared with the experimental results. The CLSVOF model calculates a more concentrated interface zone and could better describe the Al/Mg bonding interface in the stable region.
4. The transient change process of the material near the tool is predicted. Under the severe action of the tool, the Al alloy easily loses continuity. A part of the material is deposited behind the tool and the other part is migrated near the pin and makes a downward spiral movement until the tool bottom under the shear and extrusion of the pin. Finally, a stable continuous two-phase interface is formed.

## Figures and Tables

**Table 1:** Chemical composition of base materials (wt%).

Component	Al	Mg	Cu	Si	Mn	Fe	Zn	Ti	Cr
6061-T6	Bal.	1.09	0.3	0.51	0.009	0.200	0.050	-	0.13
AZ31B-H24	3.91	Bal.	0.004	0.034	0.322	0.005	0.936	-	-

## Data Availability

The data presented in this study are available on request from the corresponding author.
